# Innovative Protein Gel Treatments to Improve the Quality of Tomato Fruit

**DOI:** 10.3390/gels10010010

**Published:** 2023-12-21

**Authors:** Gabriela Luta, Daniela Balan, Maria Stanca, Ovidiu Jerca, Stefana Jurcoane, Mihaela Niculescu, Carmen Gaidau, Ioana Rodica Stanculescu

**Affiliations:** 1 Faculty of Biotechnologies, University of Agronomic Sciences and Veterinary Medicine of Bucharest, 59 Mărăsti Blvd, 011464 Bucharest, Romania; glutza@yahoo.com (G.L.); stefana.jurcoane@biotehgen.eu (S.J.); 2 Leather Research Department, Division Leather and Footwear Research Institute, Research and Development National Institute for Textiles and Leather, 93, Ion Minulescu Str., 031215 Bucharest, Romaniacarmen.gaidau@icpi.ro (C.G.); 3 Department of Analytical and Physical Chemistry, University of Bucharest, 4-12 Regina Elisabeta Bd., 030018 Bucharest, Romania; ioana.stanculescu@gmail.com; 4 “Horia Hulubei” National Institute of Research and Development for Physics and Nuclear Engineering, 30 Reactorului Str., 077125 Magurele, Romania

**Keywords:** biostimulants, bovine gelatin, keratin, lycopene, vitamin C, polyphenols, antioxidant activity

## Abstract

This study aims to establish the effect of biostimulatory protein gels on the quality of tomato. One of the most consumed vegetables, tomato (*Lycopersicon esculentum* Mill.) is a rich source of healthy constituents. Two variants of protein gels based on bovine gelatin and keratin hydrolysates obtained from leather industry byproducts were used for periodical application on the tomato plant roots in the early stage of vegetation. The gels were characterized by classical physicochemical methods and protein secondary structure was obtained by FTIR band deconvolution. After ripening, tomato was analyzed regarding its content of quality indicators (sugars and organic acids) and antioxidants (lycopene, β-carotene, vitamin C, polyphenols). The results emphasized the positive effects of the protein gels on the quality parameters of tomato fruit. An increase of 10% of dry matter and of 30% (in average) in the total soluble sugars was noted after biostimulant application. Also, lycopene and vitamin C recorded higher values (by 1.44 and 1.29 times, respectively), while β-carotene showed no significant changes. The biostimulant activity of protein gels was correlated with their amino acid composition. Plant biostimulants are considered an ecological alternative to conventional treatments for improving plant growth, and also contributing to reduce the intake of chemical fertilizers.

## 1. Introduction

Many of the modern farming techniques that require repeated soil fertilizer application for vegetable production systems are nowadays associated with increasing environmental pollution. Therefore, plant biostimulants are considered an ecological alternative to conventional treatments required to improve plant growth, and at the same time reducing the intake of chemical fertilizers. Plant biostimulants are defined as beneficial natural substances, other than fertilizers and pesticides, capable of improving the efficiency of resource use when applied to the crop in low amounts, thus promoting plant growth [1]. Various bioactive natural substances, such as animal and plant protein hydrolysates, are included in the plant biostimulants category, based on their potential to improve crop productivity. Chemically, protein hydrolysates are “mixtures of polypeptides, oligopeptides, and amino acids that are manufactured from protein sources using partial hydrolysis” [2]. Protein hydrolysates obtained by processing of residues coming from the leather industry or fish byproducts have attracted interest both for scientific and commercial reasons, due to their rich content of bioactive compounds and because these could be a sustainable and ecological solution to the problem of waste removal [3,4].

Containing amino acids and peptides, protein hydrolysates have an especially positive influence on several physiological processes, including photosynthetic activity, nutrient assimilation, and translocation in plants, and also accumulation of bioactive compounds [5]. Regarding the action mechanism of protein hydrolysates in plants, recent studies indicate that they are directly involved in stimulating carbon and nitrogen metabolism, interfering with hormonal activity. By improving absorption and accumulation of nutrients, biostimulation favors plant metabolism and provides tolerance to biotic and abiotic stress [6,7]. Thus, foliar or root application of some animal-derived protein hydrolysates led to obtaining encouraging results, such as earlier flowering and enhanced productions of fruit or flowers in strawberry [8], chili plants [9], tomato [7], and petunias [10]. Also, negative effects, such as phytotoxicity and plant growth reduction, have been reported after repeated treatments with commercial animal-derived biostimulants [11,12], which can be attributed to an incorrect use of the product’s concentration or to some inappropriate field conditions [13]. However, evaluating the safety and the efficacy as fertilizer of animal-derived biostimulants, Corte et al. [14] concluded that protein hydrolysates can be used in agricultural practices without harming human health or the environment.

Tomato (*Lycopersicon esculentum* Mill.) is one of the most-consumed vegetables worldwide, preferred not only for its organoleptic characteristics, but also as a rich source of healthy constituents. Nowadays, the daily diet is considered an important factor in preventing chronic degenerative diseases, cardiovascular disease, and different types of cancer. There are numerous studies considering that this potential effect of protection is due to a wide variety of bioactive compounds that can also be found in tomatoes: vitamins (ascorbic acid and vitamin A), phenolic compounds, and carotenoids (lycopene, zeaxanthin, β-carotene) [15,16].

Different studies reported a positive influence on tomato crops and improved production and biocompound contents after the application of biostimulants on plants [6,17], helping to reduce the use of chemical fertilizers, thus achieving the same effect.

Previous research conducted by Balan et al. [18] on tomato seedlings showed that application of protein gels increased the content of assimilatory pigments (chlorophylls and carotenoids), sugars, and phenolic compounds in the leaves when compared to untreated plants. This research assumed that development of some vigorous seedlings would result in a higher production of tomatoes with improved nutritional qualities, so the present study aimed to evaluate the biocompound accumulation in the tomato fruit under biostimulant treatment. By application on the roots, two variants of protein gels based on gelatin and keratin hydrolysates were used for treating tomato plants. After ripening, tomato fruit was harvested and analyzed for its content of nutrients known as quality indicators, such as sugars and organic acids, and the content of bioactive compounds with antioxidant activity (lycopene, β-carotene, vitamin C, and polyphenols).

There are no studies using biostimulants obtained by enzymatic hydrolysis from slaughterhouse waste in crops. The current research started from the premise that the application of the biostimulant products on tomato crops would improve the mineral nutrition of the plants and, consequently, the quality of the fruit obtained. This study is therefore focused on the effects of protein gel treatment on tomato quality and antioxidant capacity. The results show beneficial effects of the biostimulant treatments on the analyzed biocompounds in the tomatoes.

## 2. Results and Discussion

### 2.1. Protein Gel Characterization

The tested gelatin-based gels were formulated and characterized in accordance with the standards in force or in-house methods. Physicochemical attributes, composition in amino acids, and protein secondary structure of the gels were assessed.

#### 2.1.1. Physicochemical Characteristics of the Protein Gels

The physicochemical characteristics of the protein gels were performed as previously described [18] and are presented in Table 1. The data are very similar, and differences in the bloom test and viscosity are due to the addition of keratin to the gelatin-based gel.

#### 2.1.2. Amino Acid Profile

The composition of amino acid was determined for the two protein gels used (Table 2).

**Figure 1:**
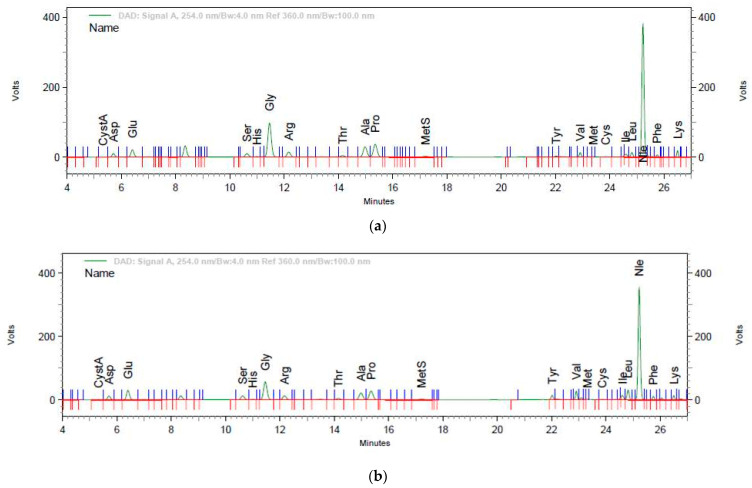
HPLC chromatograms of amino acids from the GB3 (**a**) and GB3K (**b**) products.

The amino acid profile of the GB3 product is similar with that reported by Aykin-Dincer et al. [19] for commercial bovine gelatin. The differences in the amino acid profile between the two gels are due to the addition of keratin in the GB3K product, which resulted in higher amounts of amino acids in this product. The specific chromatograms of the two products are presented in Figure 1a,b.

The GB3K product has a higher content of aspartic acid, tyrosine, valine, cysteine, leucine, and isoleucine, amino acids that play important roles in plant physiology and metabolism. Aspartic acid is an important intermediary constituent for the biosynthesis of many metabolites, such as other amino acids, nucleotides, and phytohormones, which are essential for plant growth and development, and greater resistance to abiotic stress factors [20]. A study regarding heat tolerance in perennial ryegrass showed that the exogenous application of aspartic acid had a positive result due to an enhancement in chlorophyll content and activation of enzymatic antioxidant pathways to eliminate damage caused by reactive oxygen species [21]. Amino acids aid plants in gaining better resilience to abiotic stress caused by high salinity and temperatures, drought, and heavy metals. A study made on peach seedlings highlighted the role of leucine in reducing abiotic stress generated by copper. The study showed that 10 mmol/L leucine intake significantly improved photosynthetic performance and antioxidant capacity, reduced copper accumulation, and promoted nitrogen metabolism, improving the resistance of peach seedlings to copper stress [22]. Cysteine treatments have demonstrated a favorable role in reducing the adverse effects of salinity stress on soybean plants. These treatments are effective in improving plant tolerance to salinity and may reduce the negative effect of salinity on growth and yield. For treated plants, an increase in photosynthetic pigments, enzyme activities, proline, and N, P, and K contents was noticed, while a decrease in H_2_O_2_ content was observed compared with control plants [23]. Cysteine also has a role in increasing plant immunity due to its antifungal effect, inhibiting mycelial growth [24].

#### 2.1.3. Protein Secondary Structure of Protein Gels

**Figure 2:**
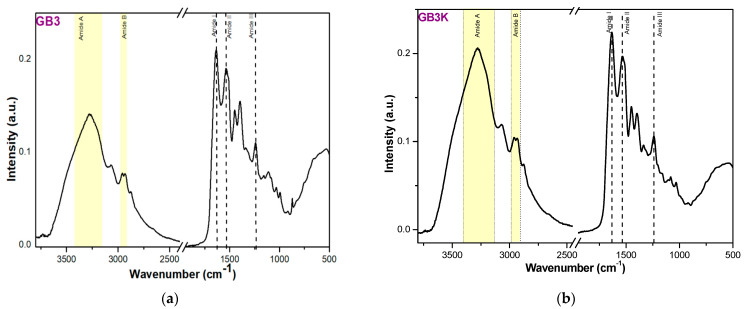
FTIR spectra of the GB3 (**a**) and GB3K (**b**) products.

Figure 2a,b present the ATR FTIR spectra of film products GB3 and GB3K, which exhibited typical amide A, B, I, II, and III bands. Amide A band is assigned to N–H stretching vibrations when the NH group of the peptide is involved in hydrogen bonding [25].

The position of amide A for the GB3K product is shifted to a lower wavenumber (3406 − 3135 cm^−1^) in comparison with amide A of the GB3 product (3423 − 3158 cm^−1^), indicating that an N–H group in an α-chain is involved in hydrogen bonding [26]. The amide B band is associated with asymmetric stretching vibration of group = C–H and –NH [27]. The amide I band in this region is attributed to C=O stretching vibrations of the amide groups coupled to the in-plane NH-bending and CN-stretching modes [28]. The amide I position depends on the conformation of protein structure and hydrogen bond. The shift to a higher wavenumber (1636 cm^−1^) for the GB3K gel is due to the presence of keratin in this product compared with GB3 gel (1634 cm^−1^) [29]. The amide II band is related to the in-plane bending vibration of N–H groups and stretching vibration of C–N groups [26]. The amide III band is a more complex band and is the result of C–N stretching vibration and N–H deformations coupled with wagging vibrations produced by CH_2_ groups from the glycine backbone and proline side-chains [30]. Lower intensity of amide III in the GB3K product showed that this product has less α-helix ordered structure than the GB3 product [31].

**Figure 3:**
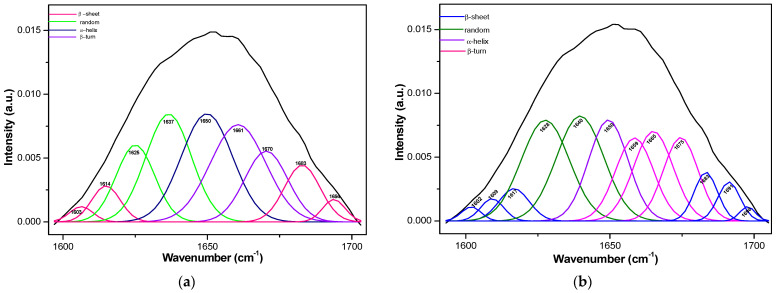
Amide I band deconvolution of the GB3 (**a**) and GB3K (**b**) products.

Information regarding protein conformation can be obtained by the deconvolution of amide I, II, and III bands. Here, we obtained this information by deconvoluting the amide I band because it is less influenced by the side-chain absorptions of the amino acids, and its intensity is higher compared to the amides II and III bands [29]. Figure 3a,b present the deconvolution of the amide I band for the GB3 and GB3K products. The GB3K product has a less-ordered secondary structure due to the addition of keratin hydrolysate, which increases the accessibility of amino acids.

Table 3 presents the content (as a percentage based on peak area) of components in the protein secondary structure of the gel products.

### 2.2. Influence of Biostimulant Treatment on the Nutritive Compound Content in the Tomato Fruit

The main effect of biostimulants is to induce physiological responses in the treated plants, affecting plant metabolism, growth, and development [32]. For the investigated tomato hybrid, biocompound content in the tomato fruit gave different responses following application of the tested protein gels.

**Figure 4:**
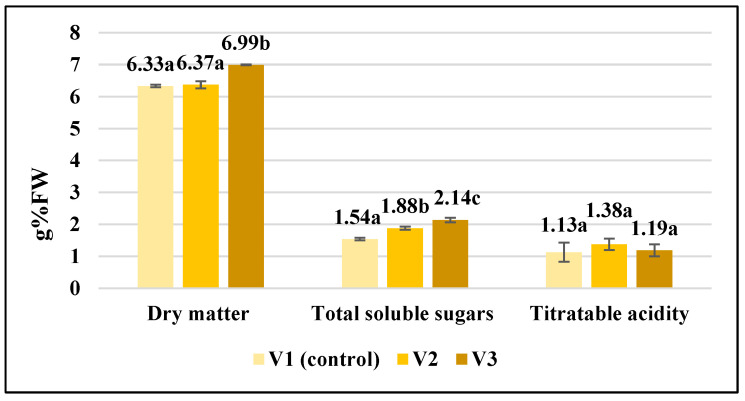
Variability of some biochemical parameters in the analyzed tomato fruit. Data are calculated as the mean of triplicate ± standard deviation; values marked with different letters show significant differences (*p* < 0.05).

The application of the biostimulants resulted in increased dry matter content in the tomato fruit, especially in the case of the V3 sample, which recorded a significant increase of 10% compared to the control variant (Figure 4). 

The same trend was noted regarding total soluble sugars, which registered an increase of 22% in the V2 sample and 39% in the V3 sample, compared to the untreated sample (Figure 4). Also, Abdelkader et al. [33] reported enhanced dry matter of tomato fruit by 14% over control (from 6.35% to 6.85%) under biostimulant treatment. On the contrary, investigating the effect of three biostimulants applied on a tomato crop, Grabowska et al. [34] found that there was no clear effect of biostimulants on the dry substance and soluble sugar amount in the tomato fruit.

Along with the sugar content, the acidity is responsible for the taste of the fruit. The research carried out in this study regarding titratable acidity showed that although there are no significant differences between the experimental variants (Figure 4), the acidity is lower (between 1.13 ± 0.30 and 1.19 ± 0.19 g% citric acid) in the tomato fruit from treated plants than in the control variant (1.38 ± 0.18 g% citric acid). Also, according to Abdelkader et al. [33], the acidity rate of the tomato fruit tended to decrease under biostimulant treatment. The low acidity of tomato fruit is involved not only in defining the taste and flavor, but also has an important contribution to food safety by destroying spoilage microorganisms [35].

### 2.3. Influence of the Biostimulant Treatment on the Content of Antioxidant Compounds in the Tomato Fruit

Tomato fruit is included in the functional food category, which positively influences the physiological and metabolic processes of the human organism [36]. The biological activity of tomatoes and their derived products have been attributed mainly to carotenoids and vitamin C, which are known as antioxidants [37].

**Figure 5:**
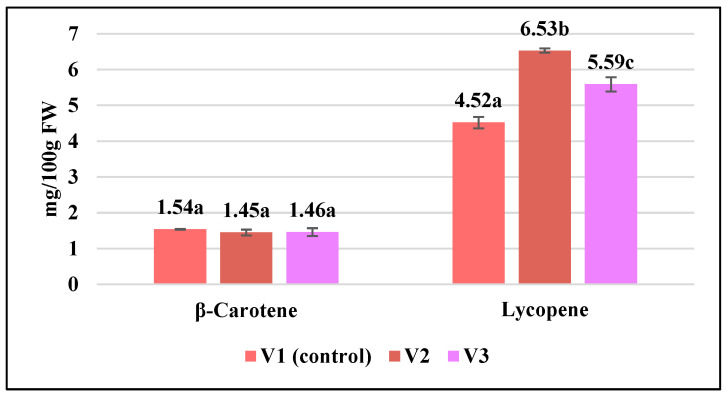
Variability of carotenoid pigments in the analyzed tomato fruit. Data are calculated as the mean of triplicate ± standard deviation; values marked with different letters show significant differences (*p* < 0.05).

The current research showed that carotenoid content in tomato fruit was modified by biostimulant application (Figure 5). 

Lycopene content in tomatoes registered a significant increase, by 1.44 times in sample V2 and by 1.23 times in sample V3, both of which received the biostimulant treatment, whereas control plants were untreated. Regarding the β-carotene content, no significant changes were noted in the case of the treated variants compared to the control. Also, Grabowska et al. [34] reported that all biostimulant treatments that were applied significantly increased lycopene content in the tomato fruit, but decreased β-carotene, regardless of the investigated tomato genotype.

Previous studies concluded that the biostimulant treatments influencing photosynthesis and secondary metabolism may also improve the chemical composition of tomato fruit by increasing total soluble solid and carotenoid contents [17,38].

**Figure 6:**
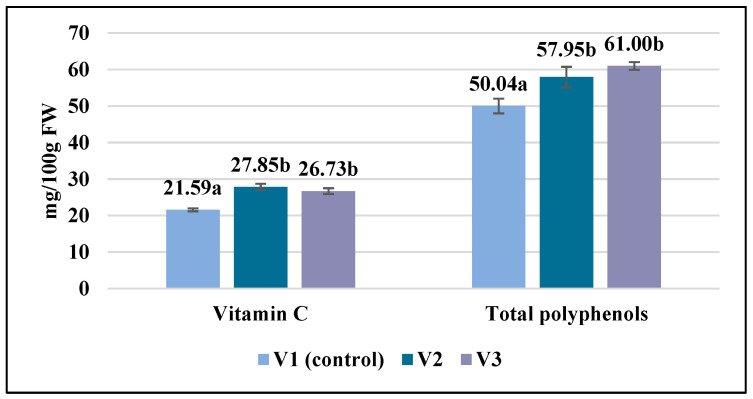
Variability of some antioxidants in the analyzed tomato fruit. Data are calculated as the mean of triplicate ± standard deviation; values marked with different letters show significant differences (*p* < 0.05).

Accumulation of vitamin C in the tomatoes was also stimulated as a result of the biostimulant treatment application (Figure 6). Thus, the vitamin C content was 1.29 times higher in the V2 sample compared to the untreated plants. No significant differences were noted between the treated samples (V2 and V3) regarding this parameter. These results are in accordance with those obtained by Tallarita et al. [17], who found that repeated biostimulant treatment led to a high accumulation of lycopene and vitamin C in tomato fruit treated with an enzymatic protein hydrolysate.

Numerous studies on this topic have concluded that the treatments with biostimulants positively impacts plant development by increasing metabolism and inducing changes in biochemical processes, so that fruit with improved quality are obtained. For instance, the research conducted by Abdelkader et al. [33] noted an increase in the total soluble solids and antioxidants, ascorbic acid, and carotenoid contents after biostimulant treatment, but it did not influence tomato fruit acidity, so tomato fruit obtained from the treated plants were tastier than those from the untreated variant.

Among the antioxidant compounds, polyphenols were also studied and significantly increased values were found in the tomato fruit after the biostimulant treatment (Figure 6). The values were between 57.95 ± 2.82 and 61.00 ± 1.05 mg GAE/100 g FW in the treated variants compared to the lowest value determined in the control (50.04 ± 2.02 mg GAE/100 g FW). Although the amount of polyphenols in the tomato fruit increased as a result of both tested protein gel applications, no significant differences were found between these experimental variants.

Antioxidants are biocompounds that induce protective effects on cells against the negative impact of reactive oxygen free radicals involved in generating oxidative stress. Investigations carried out in this field showed that the treatments with protein hydrolysates increased the formation of polyphenols, carotenoids, and antioxidant activity, thus increasing the plant tolerance to various abiotic stresses [39,40,41,42].

### 2.4. Influence of the Treatment on the Antioxidant Activity of the Tomato Fruit

Total antioxidant activity of tomato fruit is mainly a result of carotenoids, vitamin C, and phenolic compound contents.

Samples of tomato fruit from all experimental variants were tested for their potential antioxidant activity. The EC50 values were calculated to analyze all the samples regarding this parameter. The research performed in the present study indicated significant improvement in the antioxidant activity in the treated plants compared with the control plants. Significant differences were also noted between the two treatments (Table 4).

The highest antioxidant activity was registered in the tomato harvested from plants that received treatment with the GB3K gel (34.96 ± 1.10 mg/mL expressed as the EC50 value), as we expected because of the high amount of polyphenols measured in the sample (Figure 5). The lowest antioxidant capacity was noted in the tomatoes of the untreated variant, as it required a higher concentration (56.91 ± 0.49 mg/mL) to remove 50% of the DPPH free radicals.

Higher antioxidant activity after the biostimulant treatments was also reported by other studies carried out on other plant species such as lettuce [43], zucchini [44], pepper [45], and tomato [38].

Using Pearson’s correlation coefficient, a correlation study was performed to assess the relation between the antioxidant activity (expressed as EC_50_) and the amount of polyphenols, vitamin C, and carotenoids, so as to identify the major contributors to the total antioxidant capacity of the tomato fruit extracts (Table 5). The EC_50_ value found in the tomato fruit varied in inverse proportion to the amount of biochemical compounds.

All the mentioned biocompounds were found to have a strong correlation with the EC_50_ values for the tomato extracts (R = −0.8713, *p* < 0.05). Among these compounds, the strongest correlation was noted for polyphenol content (R = −0.8919, *p* < 0.05), indicating that these phytochemicals have the greatest contribution to the antioxidant capacity. The results also showed strong correlations between the antioxidant activity and the content of vitamin C (R = −0.7594, *p* < 0.05) in the tomatoes. For the total carotenoids, no significant correlation was found (−0.5788, *p* > 0.05). Previous research assessing the tomato chemical composition under biostimulant treatments showed that the treatments positively affected lycopene, but negatively affected β-carotene, so that influence on total antioxidant activity was not significantly different among the experimental treatments [34].

Overall, most studies reported that protein hydrolysates have the quality of improving plant endurance, mainly by enhancing antioxidant activity in the plant tissues, under the action of unfavorable environmental conditions [5,46].

## 3. Conclusions

The research carried out in this study showed that the application of the protein gels positively affects tomato quality by stimulating the biochemical compound accumulation in the fruit. The dry matter and sugar contents were increased as a result of the gel treatment, while the acidity was lower, contributing to a better taste of tomato fruit harvested from the plants that received treatments compared with those from untreated plants. Moreover, the applications of the tested gels revealed greater accumulation of antioxidant compounds such as vitamin C, lycopene, and polyphenols, which resulted in important health-promoting properties of the tomato fruit.

Between the two tested variants of protein gels, the most effective was the gel based on the combination of gelatin and keratin (GB3K) compared to the gel based on bovine gelatin (GB3). The biostimulant activity of protein gels was correlated with their amino acid composition. The analysis performed for gel characterization showed that addition of keratin in the GB3K product resulted in an improved content of amino acids and a less ordered secondary structure, which increases the accessibility of amino acids for the plant.

The protein gels tested in this study demonstrated stimulative effects on the tomato crop with the perspective of promoting a reduction in the use of the chemical fertilizers, thus contributing to agricultural sustainability.

## 4. Materials and Methods

### 4.1. Obtaining of Gels (Protein Hydrolysates)

Bovine hide was purchased from a local slaughterhouse in Prahova County. Sheep wool was bought from a local sheep farmer in Constanta County. Two protein gels were formulated and tested for the tomato plant treatments. The first gel was based on bovine gelatin (GB3) and the second was a mixture of bovine gelatin with keratin hydrolysate in a 1:1 ratio (GB3K).

The bovine gelatin was obtained from delimed hide, as we previously described [47]. The pelt was mixed with water in solid: liquid proportion of 1:4 and heated to 80 °C for extraction. The pH was adjusted using a solution of 1 M acetic acid, and it was controlled and adjusted every hour. After extraction, the gelatin was filtered using gauze and dried in an oven at 60 °C.

The keratin hydrolysates were obtained by alkaline hydrolysis [48]. Briefly, after washing and degreasing, the wool was shredded and then mixed with a mild alkaline solution (sodium hydroxide 2.5%) in a stainless-steel vessel. The equipment had an automatic control for temperature and a mechanical stirrer. The mixture was stirred for four hours at 80 °C. The keratin hydrolysate obtained was filtered and dried in an oven at 40 °C.

### 4.2. Determination of the Amino Acid Composition of the Protein Gels

The amino acid composition of the protein gels was performed by HPLC using an Amino Acid Analyser LC3000 (Sykam GmbH, Eresig, Germany). The instrument was equipped with a polymeric cation exchanger column and post-column ninhydrin derivatization; photometric measurement was performed at 570 nm. ChromStar 6.0 (SCPA GmbH, Bremen, Germany) chromatography software was used for processing the results. The results are reported as the mean of three determinations.

### 4.3. Determination of the Secondary Structure of the Protein Gels

FTIR measurements were performed using a Bruker Vertex 70 instrument (Bruker, Ettlingen, Germany) equipped with an ATR device. The spectra were recorded in triplicate by scanning the frequency range 4000–500 cm^−1^ with 4 cm^−1^ resolution and 32 scans by placing the powdered samples on a diamond crystal. Deconvolution of the amide I band was done to assess the degree to which the ordered structures are affected by the extraction process and to correlate it with the bioactive properties. The deconvolution parameters were settled in the spectra range of 1700–1600 cm^−1^ using a Gaussian function generated by PeakFit 4.12 software (Inpixon, Palo Alto, CA, USA). The secondary structure of the gels was obtained by using the ratio between the individual area band to the total area bands.

### 4.4. Biological Material

Research was carried out during 2022 in the experimental greenhouse at the University of Agronomic Sciences and Veterinary Medicine, Bucharest (44°26′ N latitude and 26°06′ E longitude), on Qualitet F1 hybrid tomato (*Lycopersicon esculentum* Mill.) provided by Marcoser SRL (Matca, Galati, Romania).

A monofactorial experiment was established with 3 variants of tomato plant treatments. The experimental variants were V1—untreated plants (control), V2—plants that received treatment with gelatin-based gel (GB3), and V3—plants that received treatment with gelatin-based gel activated with keratin (GB3K). The seeds were sown on 10 February 2022; the seedlings were then transplanted on 17 March 2022 into plastic pots. The plants were cultivated on the nutrient substrate Kekkila BP peat, containing optimum amounts of mineral nitrogen, phosphorus, and potassium (N:P:K ratio of 1:0.6:0.7). The application of the treatment started after the seedlings were accommodated in the pots, one week later. Three treatments were applied once every 7 days by watering the soil with diluted solutions of freshly prepared gel (30 g in 60 mL of water). On 25 April 2022, the plants from the pots were transplanted into the field. The gel treatments started a month later (25 May 2022), after the plants had adjusted to the new cultural conditions. Four treatments with the same solutions were applied once every 10 days until June 15. No treatment with other fertilizers was administered except for biostimulant applications with tested protein gels in all established variants. The control group did not receive any gel protein but only an equal amount of water.

The tomato harvest started on 25 July 2022. Random tomato samples were collected from each variant and were analyzed in the laboratory to determine the quality parameters.

### 4.5. Biochemical Analyses of the Tomato Fruit

Tomato fruit at the red-ripening stage were harvested to perform biochemical analyses. Biocompounds and the antioxidant activity were measured using appropriate analytical methods. The determinations were made in triplicate, using fresh fruit. The extractions were made according to the protocol required by the analysis method used.

Dry matter content was performed using the gravimetric method: samples had been dried to a constant mass at (105 ± 5) °C, and then the content of dry substance was calculated depending of the sample loss of weight [49].

Titratable acidity was obtained by the titration method using a solution of 0.1 N NaOH. Results were expressed as g citric acid/100 g fresh weight (FW) [50].

Vitamin C content was determined by the volumetric method [51] using titration with 0.015 M potassium iodate (KIO_3_) solution. The vitamin C was extracted in 2% hydrochloric acid (HCl) in order to prevent oxidation. The samples (10 mL) were treated with 0.2 M KI solution (5 mL), hydrochloric acid (2.5 mL), and 1% starch solution, and then titrated against KIO_3_ until the appearance of blue coloration, indicating the end point of the reaction. Results were expressed as mg/100 g FW.

Determination of total soluble sugars was performed according to the Somogyi–Nelson method described by Iordachescu et al. [52]. Acid hydrolysis (20% HCl) was performed to transform the non-reducing in reducing sugars. The resulting samples with total soluble sugars (1 mL) were heated for 15 min with alkaline copper tartrate (1 mL) and treated with arsenomolybdenic acid (1 mL), thus obtaining a colored compound (molybdenum blue). Distilled water (7 mL) was then added and the absorbance was measured at 510 nm with a ThermoHelios Alpha UV-VIS spectrophotometer. The results were expressed as g/100 g FW.

Carotenoids (lycopene and *β*-carotene) were determined using the method described by Anthon and Barrett [53]. A sample of blended tomatoes was homogenized with 100 mL of a mixture of hexane: ethanol: acetone (2:1:1); then, the two phases were separated after sonication (42 kHz) and 2 min shaking (1200 rpm). The absorbance of a sample collected from the upper phase was measured at 503 and 444 nm. Lycopene and *β*-carotene contents were calculated using Equations (1) and (2), as established by Anthon and Barrett. Results were expressed as mg/100 g FW.

C lycopene (mg/kg) = (6.95 × Abs.503 − 1.59 × Abs.444) × 0.55 × 537 × V/W(1)

C β-carotene (mg/kg) = (9.38 × Abs.444 − 6.70 × Abs.503) × 0.55 × 537 × V/W(2)

Total polyphenol content was performed using the principles of the Folin–Ciocalteu method, as described by Singleton et al. [54]. This method has been used for many years as a measure of total phenolics in natural products and can be considered a standardized method [55]. Briefly, 80% methanol samples of the extract (10 mL) were reduced with Folin–Ciocalteu reagent (1 mL) and treated with Na_2_CO_3_ reagent (8 mL, 20%). The mixture was incubated at 40 °C for 30 min. The absorbance of the obtained blue-colored compound was measured at 750 nm. The results were expressed as gallic acid equivalents (mg GAE/g FW) as the reference standard phenol.

Total antioxidant activity was assessed according to the method adapted by Brand-Williams et al. [56] after the Blois procedure [57] using the stable free radical DPPH (diphenylpicrylhydrazyl). Different concentrations of a tomato extract in 80% aqueous methanol were mixed with a 100 μM solution of DPPH in methanol and were kept 30 min in the dark. The absorbance (A) was measured at 515 nm after 30 min incubation in the dark at room temperature. The percentage of the radical scavenging activity (RSA) was calculated according to Equation (3):

% RSA = (1 − [A_sample_/A_control t = 0_])/100 (3)

A DPPH solution in 80% methanol was used as the control. The linear regression curve of the sample extracts (mg/mL) against the percentage of the radical scavenging activity was prepared and used for calculating EC_50_ for each sample. The EC_50_ parameter is defined as the concentration of sample which is required to scavenge 50% of DPPH free radicals.

### 4.6. Statistical Analyses

All measurements were replicated in three repetitions, and the results are presented as means ± standard deviation (S.D.). The significance of the effects of the protein gel applications on the biochemical characteristics was estimated by one-way ANOVA using Microsoft Excel Office 2019 for Windows. Different letters indicate significant differences between variants (*p* < 0.05). The comparison of means was calculated by the Duncan test (*p* < 0.05). Regression analysis was accomplished using Microsoft Excel Office 2019 for Windows.

## Figures and Tables

**Table 1:** Physicochemical characteristics of the used gels.

Characteristics	Gelatin-Based Gel (GB3)	Gelatin-Based Gel Additivated with Keratin (GB3K)
Dry substance (%)	6.34 ± 0.35	6.86 ± 0.38
Total ash (%)	0.26 ± 0.08	0.38 ± 0.07
Total nitrogen (%)	0.83 ± 0.06	1.02 ± 0.06
Protein content (%)	4.66 ± 0.24	5.73 ± 0.24
pH	7.34 ± 0.05	7.25 ± 0.05
Bloom test (g)	130 ± 1.15	<50 ± 1
Viscosity (mPa∙s)	1.25 ± 0.04	1 ± 0.05

**Table 2:** Amino acid composition of the gels.

Amino Acids (%)	Gelatin-Based Gel (GB3)	Gelatin-Based Gel Additivated with Keratin (GB3K)
Aspartic acid (Asp)	3.67 ± 0.01	8.27 ± 0.03
Glutamic acid (Glu)	9.76 ± 0.03	11.37 ± 0.04
Serine (Ser)	3.21 ± 0.009	3.57 ± 0.011
Glycine (Gly)	19.52 ± 0.07	17.57 ± 0.05
Histidine (His)	0.57 ± 0.002	0.62 ± 0.002
Arginine (Arg)	8.61 ± 0.02	7.24 ± 0.02
Threonine (Thr)	1.84 ± 0.005	1.76 ± 0.005
Alanine (Ala)	9.76 ± 0.003	7.75 ± 0.02
Proline (Pro)	18.94 ± 0.04	14.47 ± 0.04
Tyrosine (Tyr)	0.92 ± 0.003	2.58 ± 0.008
Valine (Val)	2.58 ± 0.07	4.03 ± 0.012
Methionine (Met)	0.29 ± 0.001	0.31 ± 0.001
Cysteine (Cys)	0.29 ± 0.001	0.98 ± 0.005
Isoleucine (Ile)	1.95 ± 0.005	2.79 ± 0.009
Leucine (Leu)	3.27 ± 0.009	5.17 ± 0.02
Phenylalanine (Phe)	2.12 ± 0.006	2.84 ± 0.008
Lysine (Lys)	3.50 ± 0.01	3.00 ± 0.009
Hydroxyproline (Hyp)	9.18 ± 0.03	5.68 ± 0.02

**Table 3:** Secondary structure (%) of gel products.

Structure	β-Sheet	Random	α-Helix	β-Turn
GB3	34.64	20.1	23.67	21.58
GB3K	14.51	35.91	14.5	35.07

**Table 4:** EC_50_ values for the experimental variants.

Variant	EC_50_ (mg/mL)
V1 (Control)	56.91 ± 0.49 ^a^
V2	44.40 ± 1.85 ^b^
V3	34.96 ± 1.10 ^c^

Data are calculated as the mean of triplicate ± standard deviation; values marked with different letters show significant differences (*p* < 0.05).

**Table 5:** The relation between analyzed biocompounds and antioxidant capacity (EC_50_) of tomatoes.

Biochemical Compounds	R (Coefficient of Correlation)	R^2^ (Determining Coefficient)	*p*
Total biochemical compounds	−0.8713	0.7592	<0.05
Total polyphenols	−0.8919	0.7955	<0.05
Vitamin C	−0.7594	0.5767	<0.05
Total carotenoids	−0.5788	0.3350	ns
Lycopene	−0.5953	0.3544	ns
β-Carotene	0.4915	0.2416	ns

ns = no significant correlation.

## Data Availability

The data presented in this study are openly available in article.

## References

[1] Du Jardin P. (2015). Plant biostimulants: Definition, concept, main categories and regulation. Sci. Hortic..

[2] Schaafsma G. (2009). Safety of protein hydrolysates, fractions thereof and bioactive peptides in human nutrition. Eur. J. Clin. Nutr..

[3] Baglieri A., Cadili V., Monterumici C.M., Gennari M., Tabasso S., Montoneri E., Nardi S., Negre M. (2014). Fertilization of bean plants with tomato plants hydrolysates. Effect on biomass production, chlorophyll content and N assimilation. Sci. Hortic..

[4] Colla G., Nardi S., Cardarelli M., Ertani A., Lucini L., Canaguier R., Rouphael Y. (2015). Protein hydrolysates as biostimulants in horticulture. Sci. Hortic..

[5] Malécange M., Sergheraert R., Teulat B., Mounier E., Lothier J., Sakr S. (2023). Biostimulant Properties of Protein Hydrolysates: Recent Advances and Future Challenges. Int. J. Mol. Sci..

[6] Colla G., Hoagland L., Ruzzi M., Cardarelli M., Bonini P., Canaguier R., Rouphael Y. (2017). Biostimulant Action of Protein Hydrolysates: Unraveling Their Effects on Plant Physiology and Microbiome. Front. Plant Sci..

[7] Sestili F., Rouphael Y., Cardarelli M., Pucci A., Bonini P., Canaguier R., Colla G. (2018). Protein Hydrolysate Stimulates Growth in Tomato Coupled with N-Dependent Gene Expression Involved in N Assimilation. Front. Plant Sci..

[8] Marfà O., Cáceres R., Polo J., Ródenas J. (2009). Animal protein hydrolysate as a biostimulant for transplanted strawberry plants subjected to cold stress. Acta Hortic..

[9] Gurav R., Nalavade V., Aware C., Vyavahare G., Bhatia S.K., Yang Y.H., Jadhav J. (2020). Microbial degradation of poultry feather biomass in a constructed bioreactor and application of hydrolysate as bioenhancer to vegetable crops. Environ. Sci. Pollut. Res..

[10] Cristiano G., De Lucia B. (2021). Petunia Performance under Application of Animal-Based Protein Hydrolysates: Effects on Visual Quality, Biomass, Nutrient Content, Root Morphology, and Gas Exchange. Front. Plant Sci..

[11] Ruiz J.M., Castilla N., Romero L. (2000). Nitrogen metabolism in pepper plants applied with different bioregulators. J. Agric. Food Chem..

[12] Lisiecka J., Knaflewski M., Spizewski T., Fraszcazak B., Kalusewicz A., Krzesinski W. (2011). The effect of animal protein hydrolysate on quantity and quality os strawberry daughter plants cv ‘Elsanta’. Acta Sci. Pol. Hortorum Cultus.

[13] Cristiano G., Pallozzi E., Conversa G., Tufarelli V., De Lucia B. (2018). Effects of an Animal-Derived Biostimulant on the Growth and Physiological Parameters of Potted Snapdragon (*Antirrhinum majus* L.). Front. Plant Sci..

[14] Corte L., Dell’Abate M.T., Magini A., Migliore M., Felici B., Roscini L., Sardella R., Tancini B., Emiliani C., Cardinali G. (2014). Assessment of Safety and Efficiency of Nitrogen Organic Fertilizers from Animal-Based Protein Hydrolysates—A Laboratory Multidisciplinary Approach. J. Sci. Food Agr..

[15] Friedman M. (2013). Anticarcinogenic, cardioprotective, and other health benefits of tomato compounds lycopene, α-tomatine, and tomatidine in pure form and in fresh and processed tomatoes. J. Agric. Food Chem..

[16] Pinela J., Oliveira B., Ferreira I., Silva L.R., Silva B. (2016). Bioactive Compounds of Tomatoes as Health Promoters. Natural Bioactive Compounds from Fruits and Vegetables.

[17] Tallarita A.V., Vecchietti L., Cozzolino E., Sekara A., Mirabella A., Cuciniello A., Maiello R., Cenvinzo V., Leone V., Caruso G. (2021). Biostimulant application improves tomato (*Solanum lycopersicum* L.) fruit yield and quality during the autumn-winter season. Res. J. Agr. Sci..

[18] Balan D., Luţă G., Stanca M., Jerca O., Niculescu M., Gaidau C., Jurcoane S., Mihalcea A. (2023). Effect of Protein Gel Treatments on Biometric and Biochemical Attributes of Tomato Seedlings in Greenhouse Condition. Agriculture.

[19] Aykin-Dincer E., Koc A., Erbas M. (2017). Extraction and physicochemical characterization of broiler (*Gallus gallus domesticus*) skin gelatin compared to commercial bovine gelatin. Poult. Sci..

[20] Han M., Zhang C., Suglo P., Sun S., Wang M., Su T. (2021). L-Aspartate: An Essential Metabolite for Plant Growth and Stress Acclimation. Molecules.

[21] Lei S., Rossi S., Huang B. (2022). Metabolic and Physiological Regulation of Aspartic Acid-Mediated Enhancement of Heat Stress Tolerance in Perennial Ryegrass. Plants.

[22] Sun M., Li S., Gong Q., Xiao Y., Peng F. (2022). Leucine Contributes to Copper Stress Tolerance in Peach (*Prunus persica*) Seedlings by Enhancing Photosynthesis and the Antioxidant Defense System. Antioxidants.

[23] Sadak M.S., Abd El-Hameid A.R., Zaki F.S.A. (2020). Physiological and biochemical responses of soybean (*Glycine max* L.) to cysteine application under sea salt stress. Bull. Natl. Res. Cent..

[24] Octave S., Amborabé B.E., Luini E., Ferreira T., Fleurat-Lessard P., Roblin G. (2005). Antifungal effects of cysteine towards *Eutypa lata*, a pathogen of vineyards. Plant. Physiol. Biochem..

[25] Rigueto C.V.T., Rossetto M., Sousa Gomes K., Loss R.A., Biduski B., Manera C., Godinho M., Brião V.B., Dettmer A., Pizzutti I.R. (2023). Steam explosion pretreatment for bovine limed hide waste gelatin extraction. Food Hydrocoll..

[26] Nagarajan M., Benjakul S., Prodpran T., Songtipya P., Kishimura H. (2012). Characteristics and functional properties of gelatin from splendid squid (*Loligo formosana*) skin as affected by extraction temperatures. Food Hydrocoll..

[27] Hidayati D., Sabiyla G.S., Prasetyo E.N., Sa’adah N.N., Kurniawan F. (2021). The Characteristic of Gelatin Extracted from The Skin of Adult and Sub-Adult Striped Catfish (*Pangasius hypophthalmus*) Using Acid-Base Pretreatment: pH and FTIR. IOP Conf. Ser. Earth Environ. Sci..

[28] Ahmad T., Ismail A., Ahmad S.A., Abdul Khalil K., Awad E.A., Akhtar M.T., Sazili A.Q. (2021). Recovery of Gelatin from Bovine Skin with the Aid of Pepsin and Its Effects on the Characteristics of the Extracted Gelatin. Polymers.

[29] Pulidori E., Micalizzi S., Koutsomarkos N., Bramanti E., Tinè M.R., Vozzi G., De Maria C., Chatzinikolaidou M., Duce C. (2023). Analysis of gelatin secondary structure in gelatin/keratin-based biomaterials. J. Mol. Struct..

[30] Jackson M., Choo L.P., Watson P.H., Halliday W.C., Mantsch H.H. (1995). Beware of connective tissue proteins: Assignment and implications of collagen absorptions in infrared spectra of human tissues. Biochim. Biophys. Acta.

[31] Kittiphattanabawon P., Benjakul S., Sinthusamran S., Kishimura H. (2016). Gelatin from clown featherback skin: Extraction conditions. LWT—Food Sci. Technol..

[32] Wally O.S.D., Critchley A.T., Hiltz D., Craigie J.S., Han X., Zaharia L.I. (2013). Regulation of phytohormone biosynthesis and accumulation in *Arabidopsis* following treatment with commercial extract from the marine macroalga *Ascophyllum nodosum*. J. Plant Growth Regul..

[33] Abdelkader M.M., Gaplaev M.S., Terekbaev A.A., Puchkov M.Y. (2021). The influence of biostimulants on tomato plants cultivated under hydroponic systems. J. Hortic. Res..

[34] Grabowska A., Kunicki E., Sękara A., Kalisz A., Jezdinský A., Gintrowicz K. (2015). The effect of biostimulants on the quality parameters of tomato grown for the processing industry. Agrochimica.

[35] Anthon G.E., Le Strange M., Barrett D. (2011). Changes in pH, acids, sugars and other quality parameters during extended vine holding of ripe processing tomatoes. J. Sci. Food Agr..

[36] Rao A.V., Agarwal S. (1999). Role of lycopene as antioxidant carotenoid in the prevention of chronic diseases: A review. Nutr. Res..

[37] Abushita A.A., Daood H.G., Biacs P.A. (2000). Changes in carotenoids and antioxidant vitamins in tomato as a function of varietal and technological factors. J. Agric. Food Chem..

[38] Rouphael Y., Cardarelli M., Bonini P., Colla G. (2017). Synergistic action of a microbial-based biostimulant and a plant derived-protein hydrolysate enhances lettuce tolerance to alkalinity and salinity. Front. Plant Sci..

[39] Luta G., Balan D., Gherghina E., Dobrin E. (2018). Effect of Foliar Bioactive Treatments on the Oxidative Stress Tolerance in Tomato Seedlings. Sci. Pap. Ser. B Hortic..

[40] Ali M.M., Jeddi K., Attia M.S., Elsayed S.M., Yusuf M., Osman M.S., Soliman M.-H., Hessini K. (2021). Wuxal amino (Biostimulant) improved growth and physiological performance of tomato plants under salinity stress through adaptive mechanisms and antioxidant potential. Saudi J. Biol. Sci..

[41] Francesca S., Cirillo V., Raimondi G., Maggio A., Barone A., Rigano M.M. (2021). A Novel Protein Hydrolysate-Based Biostimulant Improves Tomato Performances under Drought Stress. Plants.

[42] Asadi M., Rasouli F., Amini T., Hassanpouraghdam M.B., Souri S., Skrovankova S., Mlcek J., Ercisli S. (2022). Improvement of Photosynthetic Pigment Characteristics, Mineral Content, and Antioxidant Activity of Lettuce (*Lactuca sativa* L.) by Arbuscular Mycorrhizal Fungus and Seaweed Extract Foliar Application. Agronomy.

[43] Abd-Elkader D.Y., Mohamed A.A., Feleafel M.N., Al-Huqail A.A., Salem M.Z.M., Ali H.M., Hassan H.S. (2022). Photosynthetic Pigments and Biochemical Response of Zucchini (*Cucurbita pepo* L.) to Plant-Derived Extracts. Microbial, and Potassium Silicate as Biostimulants Under Greenhouse Conditions. Front. Plant Sci..

[44] Ashour M., Hassan S.M., Elshobary M.E., Ammar G.A.G., Gaber A., Alsanie W.F., Mansour A.T., El-Shenody R. (2021). Impact of Commercial Seaweed Liquid Extract (TAM^®^) Biostimulant and Its Bioactive Molecules on Growth and Antioxidant Activities of Hot Pepper (*Capsicum annuum*). Plants.

[45] Colla G., Cardarelli M., Bonini P., Rouphael Y. (2017). Foliar applications of protein hydrolysate, plant and seaweed extracts increase yield but differentially modulate fruit quality of greenhouse tomato. HortScience.

[46] González-Morales S., Solís-Gaona S., Valdés-Caballero M.V., Juárez-Maldonado A., Loredo-Treviño A., Benavides-Mendoza A. (2021). Transcriptomics of Biostimulation of Plants Under Abiotic Stress. Front. Genet..

[47] Niculescu M., Epure D., Lason-Rydel M., Gaidau C., Gidea M., Enascuta C. (2019). Biocomposites based on collagen and keratin with properties for agriculture and industrie applications. EuroBiotech J..

[48] Gaidau C., Epure D.G., Enascuta C.E., Carsote C., Sendrea C., Proietti N., Chen W., Gu H. (2019). Wool keratin total solubilisation for recovery and reintegration—An ecological approach. J. Clean. Prod..

[49] (2006). Leather–Chemical Tests-Determination of Volatile Matter.

[50] Majidi H., Minaei S., Almasi M., Mostofi Y. (2011). Total Soluble Solids, Titratable Acidity and Ripening Index of Tomato in Various Storage Conditions. Aust. J. Basic Appl. Sci..

[51] Elgailani I.E.H., Gad-Elkareem M.A.M., Noh E.A.A., Adam O.E.A., Alghamdi A.M.A. (2017). Comparison of Two Methods for The Determination of Vitamin C (Ascorbic Acid) in Some Fruits. Am. J. Chem..

[52] Iordachescu D., Dumitru I.F. (1988). Biochimie Practica.

[53] Anthon G., Barrett M.D. (2007). Standardization of a rapid spectrophotometric method for lycopene analysis. Acta Hortic..

[54] Singleton V.L., Orthofer R., Lamuela-Raventos R.M. (1999). Analysis of total phenols and other oxidation substrates and antioxidants by means of Folin-Ciocalteu reagent. Methods Enzymol..

[55] Prior R.L., Wu X., Schaich K. (2005). Standardized Methods for the Determination of Antioxidant Capacity and Phenolics in Foods and Dietary Supplements. J. Agric. Food Chem..

[56] Blois M.S. (1958). Antioxidant determinations by the use of a stable free radical. Nature.

[57] Brand-Williams W., Cuvelier M.E., Berset C. (1995). Use of a free radical method to evaluate antioxidant activity. LWT-Food Sci. Technol..

